# Tuning the electronic properties of defect-rich MoS_2_

**DOI:** 10.3762/bjnano.17.56

**Published:** 2026-06-16

**Authors:** Eric Juriatti, Martina Binninger, Carolin Schüle, Maren Zirwick, Katarina Margetic, Erika Giangrisostomi, Marcus Scheele, Heiko Peisert

**Affiliations:** 1 Institut für Physikalische und Theoretische Chemie, Universität Tübingen, 72076 Tübingen, Germany; 2 Institute Methods and Instrumentation for Synchrotron Radiation Research, Helmholtz-Zentrum Berlin für Materialien und Energie GmbH, 12489 Berlin, Germany

**Keywords:** angle-resolved photoelectron spectroscopy (ARPES), MoS_2_, phthalocyanine, X-ray absorption spectroscopy (XAS), X-ray photoelectron spectroscopy (XPS)

## Abstract

Transition metal dichalcogenides (TMDCs), including molybdenum disulfide (MoS_2_), have emerged as a promising candidate for novel semiconducting devices. However, in many cases structural defects significantly affect the electronic properties of the material. The present study utilizes angle-resolved photoelectron spectroscopy (ARPES) and surface-sensitive core-level spectroscopy (SXPS, XAS) with synchrotron radiation to investigate the interfaces between defect-rich MoS_2_ and perfluorinated cobalt phthalocyanine (CoPcF_16_). Defects were introduced in synthetic MoS_2_ bulk crystals by gentle argon and neon sputtering. Although the band structure is still visible after sputtering, especially SXPS reveals structural and electronic disturbances of the topmost MoS_2_ layer. We show how the Fermi energy in such defect-rich MoS_2_ can be tuned by the subsequent deposition of CoPcF_16_, which is verified by a shift in Fermi level for the Ne sputtered surface, under complex charge rearrangements including a charge transfer from all the substrates towards the cobalt atom of the organic molecule.

## Introduction

In the pursuit for novel semiconducting materials, the group of transition metal dichalcogenides (TMDCs), including molybdenum disulfide (MoS_2_), has emerged as a promising candidate due to their distinctive properties as layered 2D materials with broadly tunable electronic structure [1–3]. While numerous studies have focused on single-layer MoS_2_ sheets, recent research has underscored the pivotal role of multilayer TMDCs in the fabrication of ultrafast photodetectors [4–5]. To tune their electronic structure for such applications, the adsorption of organic molecules is a versatile strategy [6–7], resulting, for example, in dipole-induced doping or (complex) charge transfer [8]. Photoelectron spectroscopy (PES), X-ray absorption spectroscopy (XAS), and angle-resolved photoelectron spectroscopy (ARPES) are powerful techniques for studying interfacial electronic structures with different surface sensitivities, the orientation of molecules in a heterostructure, and the band structure in proximity to the Fermi level. Using these techniques, heterostructuring with (fluorinated) transition metal phthalocyanines (TMPcs) has been shown to modify the interfacial electronic properties of pristine TMDCs [8–10]. For the interface between CoPcF_16_ and defect-free MoS_2_, a slight doping in the TMDC was recently observed, however without strong local interactions accompanied by (integer) charge transfer [9]. A convenient side effect of this heterostructure is the strong interaction of the central Co atom of CoPcF_16_ with many metals such as Au, Ag, Cu, or Ni [11–16] such that it can be used as a probe for PES studies of TMDC layers [15]. Since interaction mechanisms depend strongly on the central metal atom and the ionization potential of the phthalocyanine, we focus specifically on CoPcF_16_ and vary the properties of the substrate.

In general, electronic defects play a crucial role in novel optoelectronic devices, as they represent, for example, traps for charge carrier transport [17]. However, it is challenging to produce nearly defect-free TMDC monolayers or multilayers. In particular, natural, exfoliated MoS_2_ crystals exhibit a variety of intrinsic defects [18]. Moreover, p- and n-type defects can be formed intentionally by sputtering with noble gases, yielding conflicting results in different reports, apparently depending on detailed experimental conditions and the source of the substrates [18–21]. Recently, a TMDC surface cleaning by noble gas ion bombardment was considered [22]. Amongst other strategies, such defects can be healed by appropriate surface modifications. For instance, the adsorption of thiol-bearing molecules has been demonstrated to remove the S-vacancies inherent to MoS_2_ grown by chemical vapor deposition [23]. It has been shown that the adsorption of different TMPcs can significantly affect surface-enhanced Raman scattering and photoluminescence properties of MoS_2_ [24–25]. Furthermore, it has been reported that the adsorption of titanyl phthalocyanine (TiOPc) might be an effective defect passivation method for MoS_2_ [26].

In the present study, we intentionally create defects in multilayered MoS_2_ by gentle ion sputtering with argon or neon and analyze the electronic structure after each treatment with PES, XAS, and ARPES. Subsequently, cobalt(II) hexadecafluorophthalocyanine (CoPcF_16_) is deposited, and the interfacial electronic structure of the defect-rich MoS_2_/TMPc heterostructure is determined. It is proposed that the electronic properties at the interface of defect-rich surfaces can be tuned by the interaction with the deposited molecule.

## Experimental

All experiments were conducted either at our home laboratory setup or at the LowDosePES endstation of the PM4 beamline (BESSY II, Helmholtz-Zentrum Berlin, Germany) under ultrahigh vacuum (UHV) conditions (base pressures: at our home laboratory 8 × 10^−10^ mbar and 1 × 10^−10^ mbar at the LowDose PES endstation). The spectrometer at the home laboratory is equipped with a monochromatized Al Kα radiation source (XR 50 M, SPECS GmbH) and a hemispherical analyzer (Phoibos 150, SPECS). The energy was calibrated to the binding energies of Au 4f_7/2_ (84.00 eV), Ag 3d_5/2_ (368.21 eV) and Cu 2p_3/2_ (932.63 eV) using Ar-ion sputtered foils of Au, Ag, and Cu (Goodfellow Cambridge Ltd.). At the LowDosePES endstation the samples were investigated by XAS and (angle-resolved) PES using an angle-resolved time-of-flight detector (ARTOF). For XAS, the total electron yield of the sample current was measured. The energy resolution for the Co L edges was 263 meV at a photon energy of 790 eV. For photoemission at excitation energies of 900, 500, 370, 300, 230, and 75 eV, the energy resolutions were 592, 230, 156, 114, 118, and 66 meV, respectively. A sputtered gold crystal was used for energy calibration. After every change of energy, X-ray photoelectron spectra (XPS) of Au 4f, calibrated to 84.00 eV, or the Au Fermi edge, calibrated to 0.00 eV, were acquired.

Synthetic molybdenum disulfide crystals (2D Semiconductors, Phoenix, AZ, USA) were attached to the sample holders using silver conductive paint. To ensure cleanliness, the crystals were cleaved in situ by scotch tape. By the absence of carbon and oxygen signals in the XPS, a contamination-free surface could be confirmed. The samples were sputtered with a IQE 10 ion source (SPECS GmbH, Berlin, Germany) using Ar and Ne gas (purity: 4.5, Linde plc, Dublin, Ireland and Westfalen AG, Münster, Germany). The pressure in the vacuum chamber was maintained at a constant level of 5 × 10^−5^ mbar during the sputtering process. The kinetic energy of the ions was set to 500 eV at the synchrotron facility and 250 eV at the home laboratory. The sputter time was varied. Subsequently, CoPcF_16_ (95%, BLD Pharmatech LTD., Shanghai, China) was deposited in a separate chamber at temperatures of 450 °C and a pressure of approximately 1 × 10^−7^ mbar using a Knudsen cell. Via a quartz crystal microbalance, the deposition rate was determined as 0.1–0.2 nm·min^−1^. The nominal layer thickness of the deposited CoPcF_16_ molecule on the MoS_2_ substrate was calculated by comparing core level intensities of the overlayer (C 1s) to the intensity of the substrate related Mo 3d signal. The photoionization cross sections were taken from Yeh and Lindau [27], and the inelastic mean free path was calculated using the formula given by Seah and Dench [28] for organic molecules.

The XPS spectra were fitted using Unifit software (Unifit Scientific Software GmbH, Leipzig, Germany). The Voigt profile as a convolution of Gaussian and Lorentzian line shapes and a Shirley background was chosen.

The ARPES data were processed using IGOR Pro software, subtracting a detector image for intensity correction. The 2D images shown in this study were extracted from 3D images in Γ–K and Γ–M directions, that is, the most important points of high symmetry within the reciprocal space.

## Results and Discussion

### Effects of sputtering on the MoS_2_ surface and interface properties of MoS_2_/CoPcF_16_

**Figure 1:**
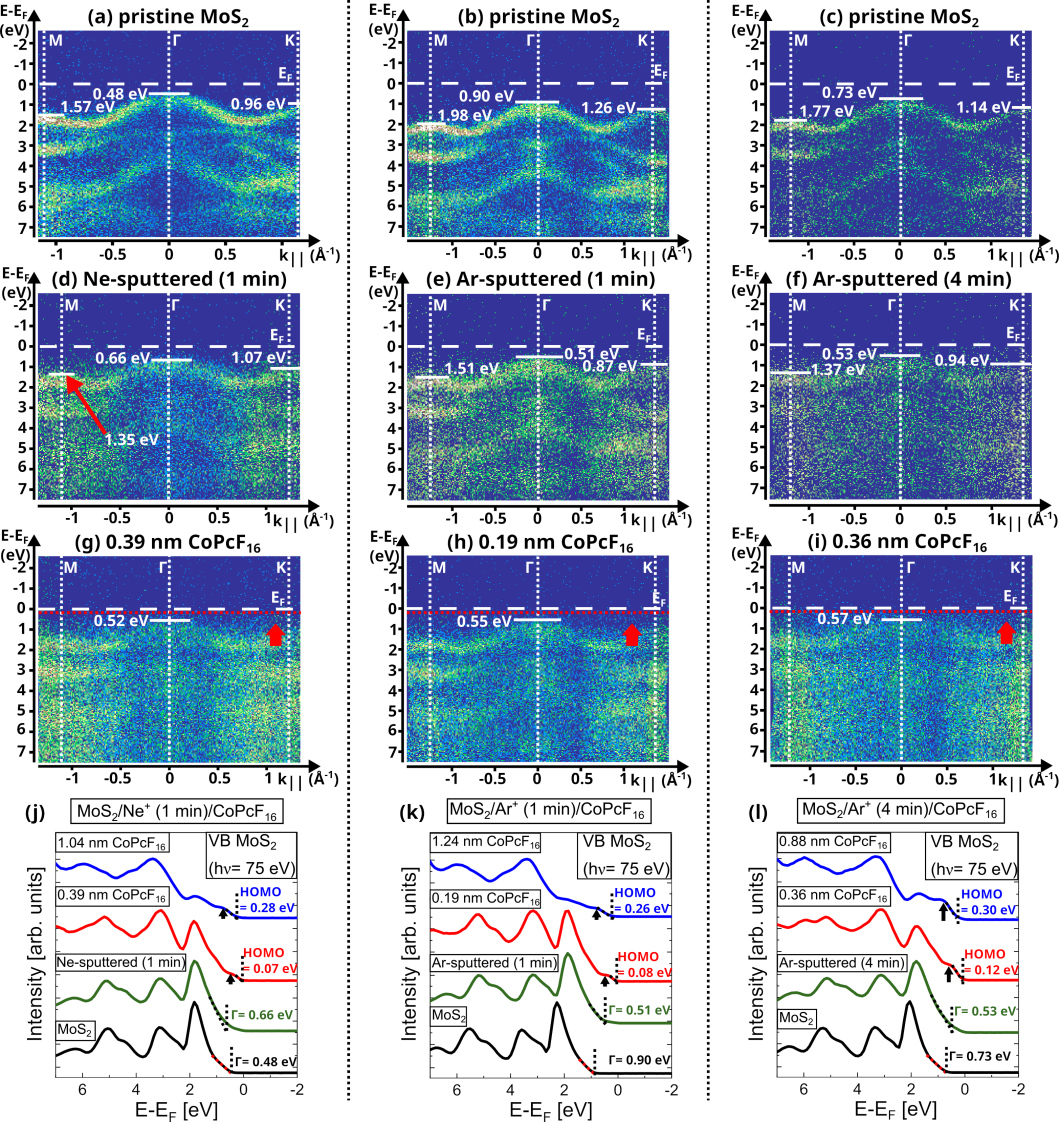
ARPES band structure of MoS_2_ before (a–c) and after sputtering (d–f) and the subsequent deposition of a monolayer film of CoPcF_16_ (g–i), and the integrated valence band structures (j–l) measured at an excitation energy of *h*υ = 75 eV.

In Figure 1, the variations of the position of the Fermi level of MoS_2_ upon Ar^+^ and Ne^+^ ion bombardment and subsequent deposition of CoPcF_16_ in the monolayer range are monitored using ARPES M–Γ–K maps of three different experiments, namely, Ne ion sputtering for 1 min (Figure 1a,d,g), Ar ion sputtering for 1 min (Figure 1b,e,h), and Ar ion sputtering for 4 min (Figure 1c,f,i). A low energy of 500 eV was used to avoid more severe radiation damage of MoS_2_ (for further details, e.g., the corresponding *k_x_*–*k_y_* maps, see Figure S1 in Supporting Information File 1). To ascertain the valence band maximum as the energy of the highest occupied state, the Fermi edge of a sputtered gold foil was measured prior to each experiment to calibrate the Fermi level to 0.00 eV. The exact position of the valence band maximum was determined utilizing the integrated band maps at the located Gamma point as shown in Figure 1j–l.

In general, energetic shifts of core levels or valence band features in photoemission can be attributed to different origins including a change of the electron density at the considered atom, different screening of the remaining photo-hole, or shifts of the reference (Fermi) level. Screening effects by additional adsorbates depend strongly on the dielectric constant, and the distance would cause a lowering of the binding energy. The magnitude might be estimated to maximal 0.1–0.2 eV for an atom directly at the interface to an organic molecule [29]. Since the observed energetic shifts are similar for different substrate-related core levels and the signal is not limited to the uppermost atoms only, we discuss them in the following in the frame of a shift of the reference (Fermi) level due to band bending. This change in the Fermi level is referred to as (relative) p- or n-doping.

It is important to note the varying initial Fermi level positions at 0.48, 0.90, and 0.73 eV above the valence band maximum for the three experiments (Figure 1a–c). Assuming an (indirect) band gap of 1.23 eV [30–31], this indicates initial p-doping for the first sample, as well as n-doping for the other two samples. The ion bombardment causes energetic shifts to these initial Fermi level positions without compromising the integrity of the band structure. In experiment one, the sputtering with Ne, the Gamma point shifts to 0.7 eV (Figure 1d), now indicating a slight n-doping. In contrast, after Ar sputtering for 1 or 4 min, the Gamma point is located about 0.5 eV below *E*_F_, which corresponds to a slight p-doping (Figure 1e,f; integrated spectra: Figure 1k,l).

Subsequent deposition of roughly a monolayer of CoPcF_16_ on the sputtered substrates causes further energetic shifts, which are small but above the energetic resolution of our experiment, after which the valence band maximum at the Gamma point (Figure 1g–i) lies between 0.5–0.6 eV in all three cases. In addition to the band structure derived from the MoS_2_ substrate, new non-dispersive bands emerge below the Fermi level, marked by the red dotted lines and arrows in Figure 1g–i and best visible in the integrated valence band spectra (Figure 1j–l, red curves) highlighted by black arrows. These effects are attributed to the molecular orbitals of CoPcF_16_ at the interface. In accordance with this interpretation, an even more pronounced manifestation of such additional energy levels is observed for thicker molecular coverages, exemplified in Figure 1j–l with the blue curves (and in more detail in Figure S1, Supporting Information File 1). With increasing molecular deposition, their position shifts to higher energies until reaching the position of the HOMO of the free CoPcF_16_ molecule at approximately 0.3 eV below the Fermi level.

The results presented in Figure 1 are largely consistent with previous studies on the subject of sputtering MoS_2_ surfaces: Both Ne^+^ and Ar^+^ ion bombardment of MoS_2_ have been reported to cause changes in the electronic structure [19–20]. However, depending on the gas and the precise sputtering conditions, the mechanisms are somewhat different and the results not always comparable. It was reported that Ar sputtering at high energies (10 keV) yields a p-doped substrate accompanied by the formation of Mo^0^ islands [19]. For Ne-sputtered MoS_2_, the literature is much less clear. For 1 keV Ne^+^ bombardment, Lince et al. [20] reported slight shifts of the Mo 3d core levels towards lower binding energies and more intense shifts towards higher binding energies for the S 2p signals, precluding any conclusions on possible doping. Further results in the context include the observation of distinct defects in the microstructure already upon low ion dosing [21,32], as well as significant alterations of the Mo/S atomic ratio [19–21].

We ascribe the variations of the initial doping level in our samples to the presence of step edges and/or non-detectable traces of adsorbates. However, considering that even low doses of ion sputtering can result in a significantly higher number of defects, we presume that these sample-dependent fluctuations do not relevantly affect the qualitative trends found after sputtering and the deposition of CoPcF_16_.

Overall, the results of this study indicate that sputtering is a viable method for shifting the Fermi level in MoS_2_, thereby affording slight p- (Ar) or n- (Ne) doping. In addition, the thickness-dependent results after deposition of CoPcF_16_ indicate a surface interaction with the sputtered MoS_2_, which will be expanded in the following sections.

### Interaction mechanism at the interface between the sputtered MoS_2_ substrate and CoPcF_16_

In the case of TMPCs, two distinct interaction channels are possible involving the macrocycle and/or the central metal atom [11]. First, the interaction via the Co atom of CoPcF_16_ will be examined utilizing XAS at the Co L_2,3_ edge. Co L_2,3_ edge XAS is sensitive to the interfacial electronic structure and provide information on molecular orientation. Co L_2,3_ spectra of CoPcF_16_ exhibit a typical multiplet structure, analogously to [11,33], with three primary features:, namely, A (polarized orthogonal to the molecular plane, *z*), B1, and B2 (polarized within the molecular plane, *xy*).

**Figure 2:**
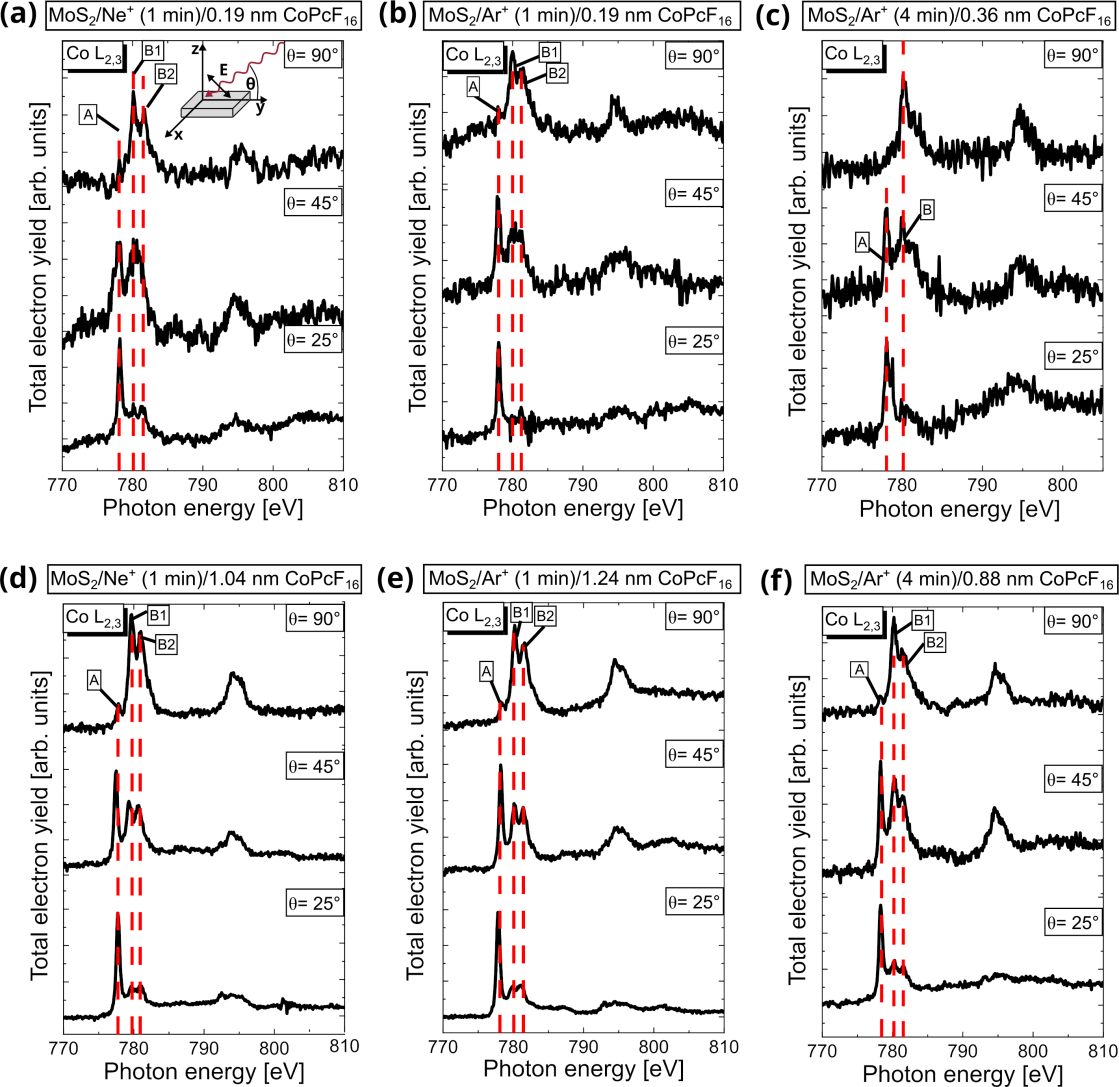
Co L_2,3_ XAS spectra of CoPcF_16_ coverages in the mono- or multilayer range on Ne^+^ or Ar^+^ sputtered MoS_2_.

The Co L_2,3_-XAS results are interpreted using p-polarized synchrotron light in Figure 2 in the context of these three features, which are highlighted by dashed red lines. For all three substrates and approx. monolayer CoPcF_16_ coverage (see Figure 2a–c), the largest intensity is observed at grazing beam incidence (θ = 25°) at feature A. Changing the angle of incidence to 45° and 90° strongly diminishes the intensity of feature A, while the features B1 and B2 gain relative intensity.

Increasing the CoPcF_16_ thickness has no major effects on the Co L_2,3_ XAS results for the samples sputtered with Ne^+^ (1 min) and Ar^+^ (1 min) (compare Figure 2a vs Figure 2d, and Figure 2b vs Figure 2e, respectively). In general, these spectra are similar to bulk-like spectra, suggesting the absence of strong interfacial interactions. In contrast, for the more defect-rich, 4 min Ar^+^-sputtered MoS_2_ substrate, we detect significant changes of the spectral shape. Most notably, the relative intensity of B2 at normal incidence (90°) is substantially weaker than B1 for 2–3 layers of CoPcF_16_ (Figure 2f) and almost completely vanishes for a monolayer coverage (Figure 2c).

These results indicate almost planar, flat lying CoPcF_16_ molecules on the MoS_2_ surface, enabling generally strong interactions of the central Co atom of CoPcF_16_ with the substrate. Such interactions can include a redistribution of the d electrons, charge transfer, or even the formation of new states in the case of strong chemisorption [11]. The latter was observed for CoPc on reactive metal surfaces like Ag or Ni [11]. However, in the present case, such interactions exclusively manifest for the heavily sputtered Ar^+^ (4 min) sample. This behavior is more in line with the previously documented interactions of CoPcF_16_ with relatively inert metals including oxygen-terminated copper or graphene-covered Ni [11,33–34]. Based on these similarities, we suggest that charge transfer and/or electron redistribution at the Co atom in the vicinity of such defect-rich MoS_2_ is responsible for our observations in Figure 2c,f.

**Figure 3:**
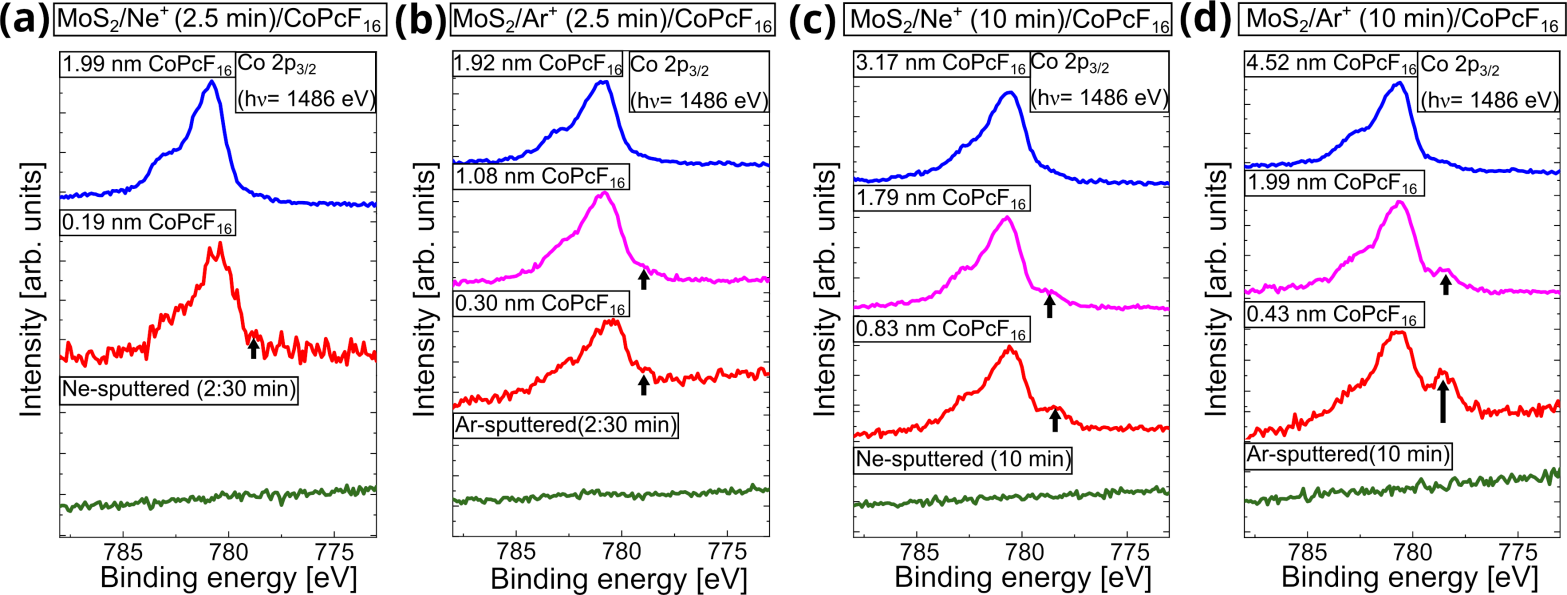
Co 2p XPS spectra before and after the deposition of mono- and multilayer films of CoPcF_16_ after (a) 2.5 min Ne sputtering (b) 2.5 min Ar sputtering, (c) 10 min Ne-sputtering, and (d) 10 min Ar sputtering.

Figure 3 substantiates the study of the interaction between CoPcF_16_ and heavily sputtered MoS_2_ surfaces. We recorded with the home-lab instrument Co 2p XPS spectra of MoS_2_ surfaces sputtered with Ne or Ar gas for 2.5 or 10 min, and after deposition of a gradually increasing number of CoPcF_16_ layers. Consistent with the XAS results in Figure 2, an additional peak was found at 778–779 eV (see black arrows in Figure 3) for a monolayer coverage, which decreases in intensity as the thickness rises. The peak is particularly prominent for the longest sputtering times (Figure 3c,d), corroborating that strong Co–MoS_2_ interactions require a high defect density. A similar peak for CoPcF_16_ at interfaces with metal substrates like Au and Cu and also WSe_2_ was found, which was ascribed to a reduced Co species resulting from the charge transfer from the substrate to the central metal atom of the organic molecule [15,35–36]. For the unsputtered MoS_2_ substrate, this charge transfer was neither observed in the XAS Co L_2,3_ edges nor in the Co 2p photoemission spectra [9,37]. This demonstrates that the high concentration of surface defects is crucial for such an interaction. For multilayer coverage, for example, when the effect of the substrate becomes negligible, the Co 2p spectra exhibit the typical multiplet structure for Co^2+^ [11,14,38–39].

**Figure 4:**
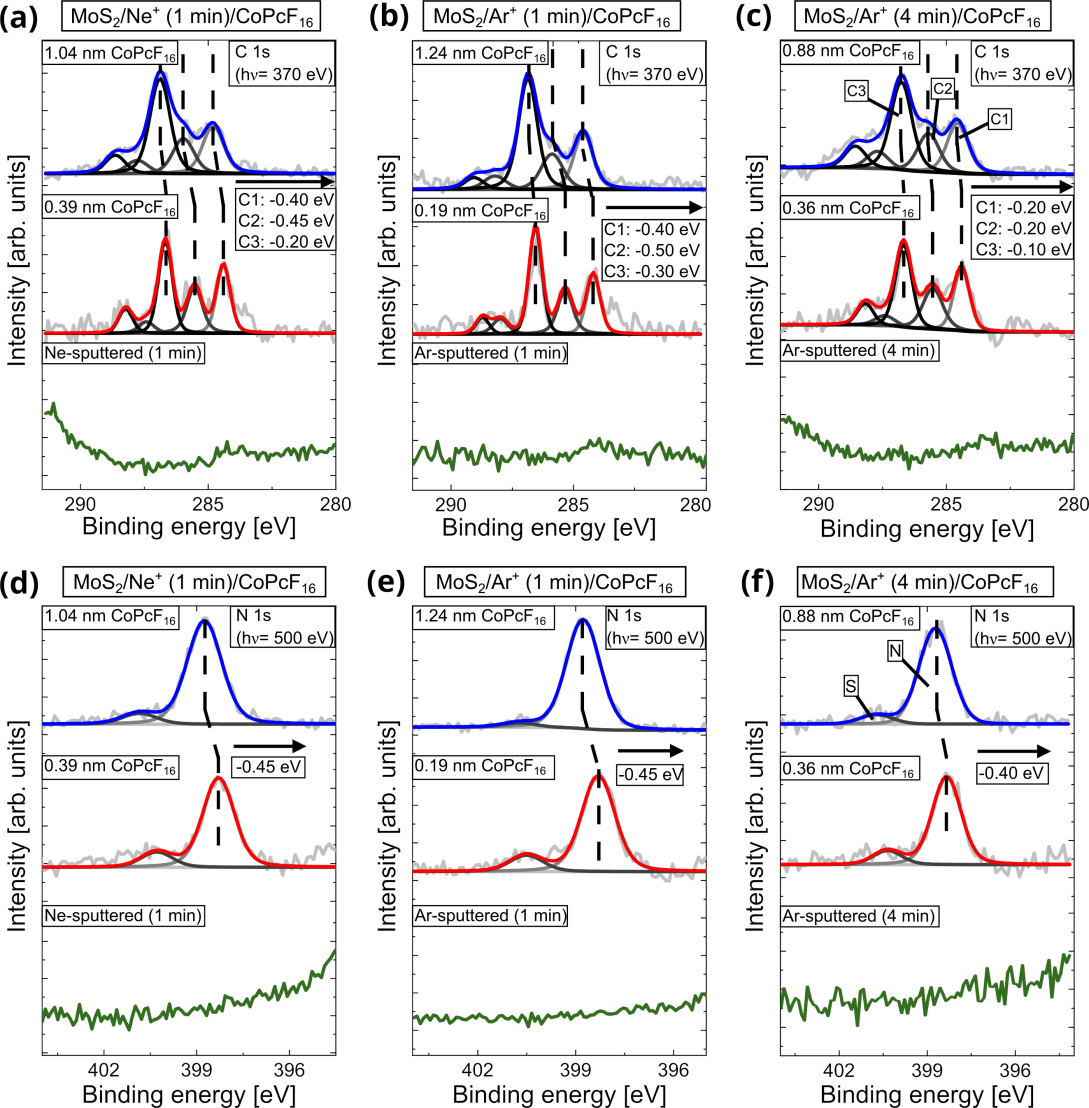
Molecule-related C 1s (a,b,c) and N 1s (d,e,f) core level spectra on differently sputtered MoS_2_ surfaces. The absence of additional interface peaks indicates the absence of strong, local interactions.

Figure 4 presents the results into an alternative interaction mode between the sputtered MoS_2_ surface and CoPcF_16_ by XPS, namely, that of the carbon- and nitrogen-rich macrocycle. In summary, the C 1s (Figure 4a–c) and N 1s spectra (Figure 4d-f) following the deposition of CoPcF_16_ of varying thicknesses onto MoS_2_ substrates, sputtered with Ne^+^ or Ar^+^ and varying durations up to 4 min, exhibit no signs of strong interactions like bond breaking or local charge transfer to a particular atom. The molecules remain intact, and possible interactions of the macrocycle are most likely limited to complex (bidirectional) charge rearrangements at the interface, as observed for many similar systems [35,40–42]. The shift towards lower binding energies between the free molecule, indicated by the thicker film (blue line), and the interface (red line) can be mostly attributed to commonly observed screening of the photohole at the interface [9–10,43]. For details on the fitting procedure underlying this conclusion, refer to Supporting Information File 1.

### Surface sensitivity of substrate-related energetic shifts

**Figure 5:**
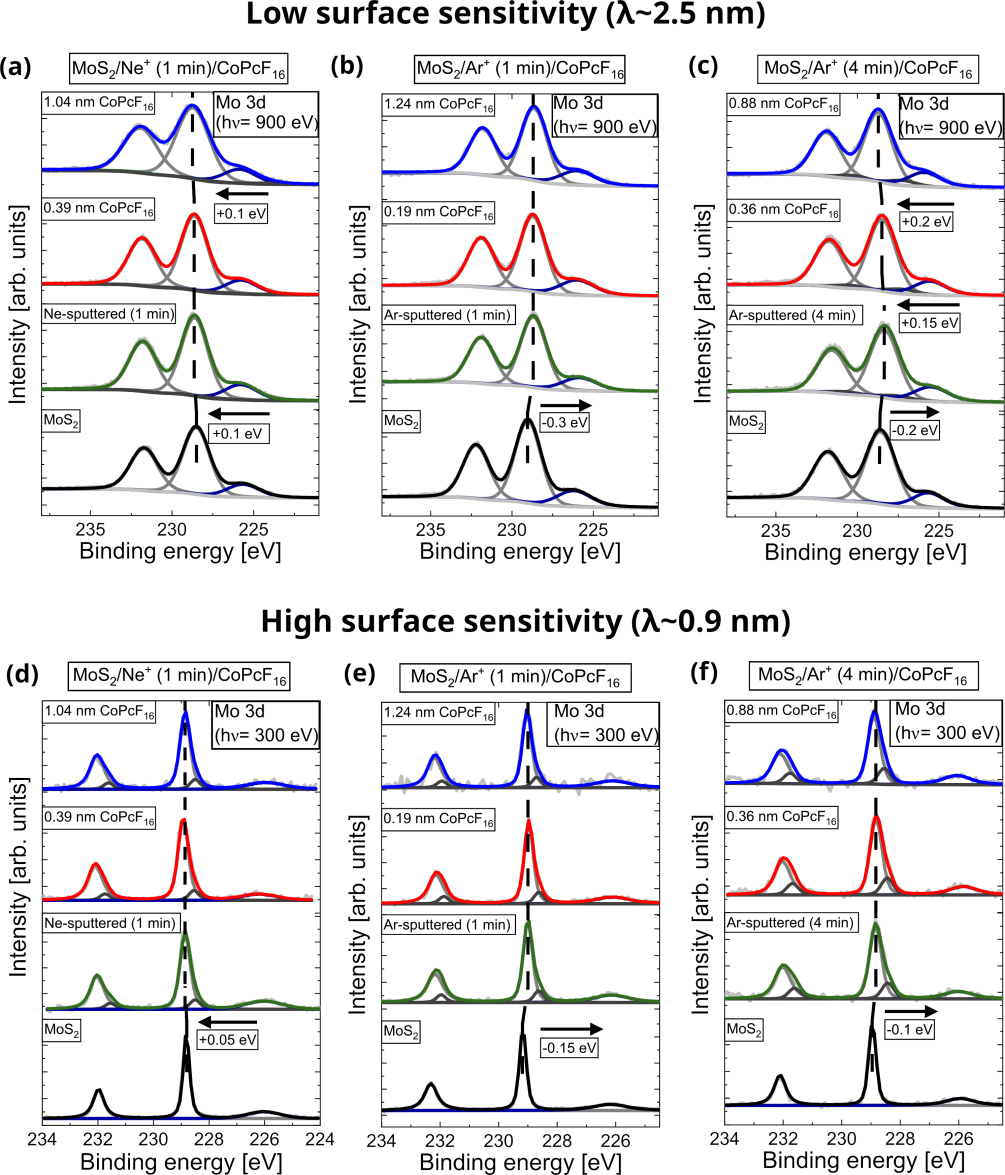
XPS measurements of the Mo 3d core levels of MoS_2_ following the sputtering with Ar^+^ and Ne^+^ and the deposition of CoPcF_16_ measured at excitation energies of 900 and 300 eV, resulting in different inelastic mean free paths, λ, of the detected photoelectrons and thus a different surface sensitivity. Additional peaks after sputtering (dark grey lines) are attributed to Mo^0^ species. Shifts towards higher (lower) binding energies are indicated by black arrows.

In Figure 5, we use Mo 3d core level spectra (accompanied by the S 2p spectra in Supporting Information File 1, Figure S2), measured with excitation energies of 900 or 300 eV, to study how far the effects of sputtering and CoPcF_16_ deposition extend into the bulk of the MoS_2_ substrate. The corresponding mean free paths, λ, calculated for the two energies according to [28] for inorganic materials, are 2.5 nm (“low surface sensitivity”) and 0.9 nm (“high surface sensitivity”). All spectra consist of Mo 3d_5/2_ and Mo 3d_3/2_ doublets at binding energies of 228–229 eV and 232–232.5 eV, respectively, and the S 2s peak at 225.5–226.5 eV. All fit parameters are summarized in Tables S4 and S5 (Supporting Information File 1).

With low surface sensitivity, the shifts of the Mo 3d (Figure 5a–c) and S 2p core level spectra (Supporting Information File 1, Figure S2) after sputtering and molecular deposition (Figure 5a–c) are in reasonable agreement with the shifts of the valence band spectra in Figure 1 and corroborate our previous interpretation as p- or n-doping. A further consistence between these two experiments is found in the partial reversal of the sputter-induced energetic shifts by a subsequent deposition of CoPcF_16_. Monitoring the Mo/S ratios (Supporting Information File 1, Table S7) yields no significant difference inflicted by sputtering, indicating that these actions do not change the composition of the bulk.

In contrast, with high surface sensitivity, we find an increasing Mo/S ratio (Supporting Information File 1, Table S7; especially for Ar^+^ (4 min) sputtering) and the appearance of an additional Mo species, which we attribute to Mo^0^ (Figure 5d–f) [19–20]. These findings are consistent with recent microscopic studies that have demonstrated structural surface changes due to sputtering [21,32], as well as reports on the formation of Mo^0^ after sputtering at higher energies or with relatively high doses of various noble gases [19–20,22]. The effect of Ar^+^ sputtering on the Mo/S ratio is larger than for Ne^+^ for the same sputtering time. These results indicate a severe degradation of the topmost layer of sulfur atoms during sputtering.

It is noted that, based on the mean free path, one would expect similar surface sensitivities for the valence band spectra in Figure 1 (λ = 5–10 Å) [44–46] and the core level spectra in Figure 5d–f. However, the core level shifts seen with high surface sensitivity, although qualitatively identical, are quantitatively less pronounced than the changes in the valence band. We suggest that this could be an indication that these uppermost fragments do not determine the electronic structure depicted in Figure 1. The sharp features shown therein may originate to a large extent from deeper layers, broadened and attenuated due to scattering at the topmost atoms. Contributions from the top layer appear as background intensity in Figure 1 and do not contribute to the well-defined bands due to the lack of periodicity over larger distances.

However, an exact agreement between the core level shifts acquired with low surface sensitivity and the valence band shifts in Figure 1 cannot be expected either since, for λ = 2.5 nm, the topmost layer still contributes significantly to the spectra.

## Conclusion

By this study, a deeper understanding of defect-rich MoS_2_ heterointerfaces is given. It has been demonstrated that by sputtering with different noble gases (Ar and Ne), the structure and electronic properties of the top layer are significantly disturbed. Energetic shifts in valence band and core level spectra reveal p- or n-type doping effects, most likely due to the presence of sulfur vacancies and metallic Mo. Nevertheless, the deposition of CoPcF_16_ affects the position of the Fermi energy, which illustrates that a tuning of electronic properties is also possible in the case of MoS_2_ substrates with a high density of surface defects. The molecules remain intact and a local charge transfer to the Co atom is observed for a small fraction of molecules only, especially for high defect concentrations (4 min Ar^+^). Most likely complex (bidirectional) charge rearrangements occur at the interface.

## Supporting Information

File 1 Additional figures and tables.

## Data Availability

Data generated and analyzed during this study is available from the corresponding author upon reasonable request.
