# Supplementary material for: Tuning the electronic properties of defect-rich MoS2
Source: Beilstein J Nanotechnol. 2026 Jun 16;17:796–805. doi: 10.3762/bjnano.17.56 (PMC13284750; doi:10.3762/bjnano.17.56)
Supplement: File 1 — Additional figures and tables. [file Beilstein_J_Nanotechnol-17-796-s001.pdf]

BEILSTEIN JOURNAL OF NANOTECHNOLOGY

Supporting Information

for

Tuning the electronic properties of defect-rich MoS_2

Eric Juriatti, Martina Binninger, Carolin Schüle, Maren Zirwick, Katarina Margetic, Erika Giangrisostomi, Marcus Scheele and Heiko Peisert

Beilstein J. Nanotechnol. **2026,** *17,* 796–805. doi:10.3762/bjnano.17.56

Additional figures and tables

License and Terms: This is a supporting information file under the terms of the Creative Commons Attribution License (https://creativecommons.org/licenses/by/4.0). Please note that the reuse, redistribution and reproduction in particular requires that the author(s) and source are credited and that individual graphics may be subject to special legal provisions.
The license is subject to the *Beilstein Journal of Nanotechnology* terms and conditions: (https://www.beilstein-journals.org/bjnano/terms)

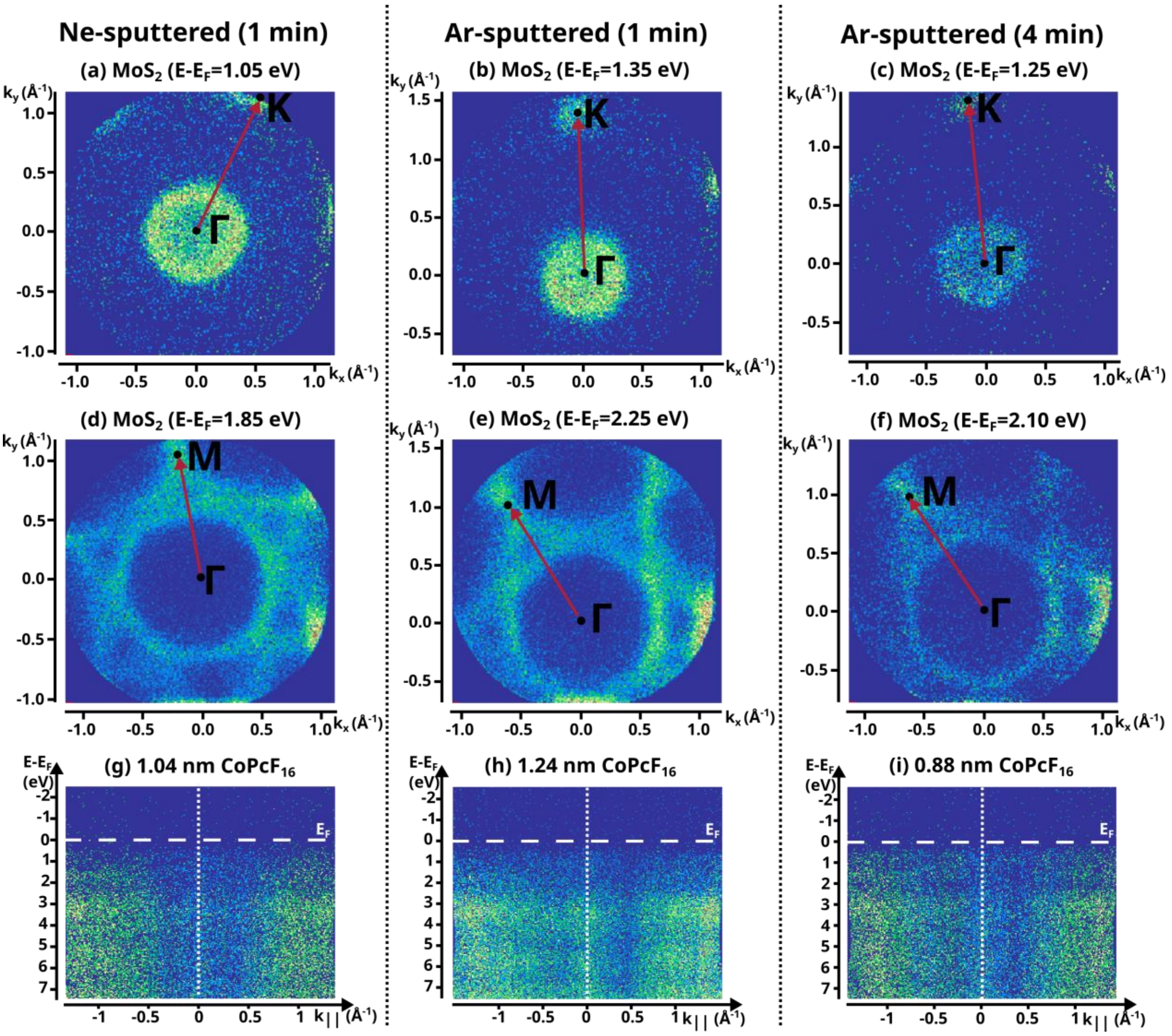

Figure S1: kx-ky-maps at selected energies showing K (a-c) and M (d-f) for the three pristine MoS_2 samples and ARPES band structures of the multilayer $CoPcF_{16}$ coverages (g-i) on given substrates.

The fitting of the C 1s and N 1s spectra shown in Figure 4 was performed using established models for perfluorinated phthalocyanines [1]. The C 1s spectra consist of three individual carbon components, representing C-C (C1), C-N (C2) and C-F (C3) bonds, accompanied by satellite peaks (S1, S2, S3) at higher binding energies. The intensity ratio C1+S1/C2+2/C3+S3 is in good agreement with the stoichiometry of $CoPcF_{16}$ (see Table S1). The N 1s spectra were fitted with a single main nitrogen peak (N) and an additional satellite (S) at higher binding energies (see Table S2). For all spectra, the fitting model was essentially independent of the

film thickness and the underlying substrate. The different energetic shifts of the individual carbon components and the N 1s level, indicated by broken lines in Figure 4, may hint to a different electron distribution in the molecule at the interface compared to the bulk, but can also be explained by site-dependent screening effects [2,3].

Table S1: Fit parameters for the C 1s spectra shown in Figure 4.

**a) Ne^+ (1 min) + 0.39 nm $CoPcF_{16}$**	Position [eV]	Gaussian width [eV]	Lorentzian width [eV]	rel. area [%]
C1	284.40	0.55	0.15	26.6
S1	285.75	0.55	0.15	0.5
C2	285.53	0.54	0.30	21.7
S2	287.42	0.54	0.30	5.4
C3	286.67	0.56	0.14	36.1
S3	288.23	0.56	0.14	9.6
**Ne^+ (1 min) + 1.04 nm $CoPcF_{16}$**				
C1	284.80	0.80	0.30	22.8
S1	286.01	0.80	0.30	1.1
C2	285.97	0.81	0.30	17.1
S2	287.78	0.81	0.30	6.5
C3	286.89	0.76	0.30	44.0
S3	288.63	0.76	0.30	8.5

**b) Ar^+ (1 min) + 0.19 nm $CoPcF_{16}$**	Position [eV]	Gaussian width [eV]	Lorentzian width [eV]	rel. area [%]
C1	284.42	0.55	0.15	24.1
S1	285.63	0.55	0.15	0.5
C2	285.52	0.52	0.30	21.3
S2	288.06	0.52	0.30	7.5
C3	286.68	0.55	0.10	40.3
S3	288.76	0.55	0.10	6.3
**Ar^+ (1 min) + 1.24 nm $CoPcF_{16}$**				
C1	284.81	0.70	0.30	24.0
S1	286.01	0.70	0.30	0.5
C2	286.04	0.83	0.30	17.2
S2	288.27	0.83	0.30	6.5
C3	286.98	0.69	0.30	46.8
S3	289.09	0.69	0.30	5.0

**c) Ar^+ (4 min) + 0.36 nm $CoPcF_{16}$**	Position [eV]	Gaussian width [eV]	Lorentzian width [eV]	rel. area [%]
C1	284.40	0.65	0.15	26.0
S1	285.75	0.65	0.15	0.8
C2	285.53	0.75	0.30	22.3

S2	287.42	0.75	0.30	5.6
C3	286.67	0.65	0.18	35.9
S3	288.17	0.65	0.18	9.5
**Ar^+ (4 min) + 0.88 nm $CoPcF_{16}$**				
C1	284.56	0.80	0.30	22.9
S1	286.16	0.80	0.30	0.7
C2	285.70	0.83	0.30	18.1
S2	287.70	0.83	0.30	8.1
C3	286.76	0.80	0.30	40.2
S3	288.56	0.80	0.30	10.1

Table S2: Fit parameters for the N 1s spectra shown in Figure 4.

**d) Ne^+ (1 min)+ 0.39 nm $CoPcF_{16}$**	Position [eV]	Gaussian width [eV]	Lorentzian width [eV]	rel. area [%]
N	398.29	1.04	0.30	85.4
S	400.29	1.04	0.30	14.6
**Ne^+ (1 min) + 1.04 nm $CoPcF_{16}$**				
N	398.75	1.16	0.30	90.6
S	400.85	1.16	0.30	9.4

**e) Ar^+ (1 min) + 0.19 nm $CoPcF_{16}$**	Position [eV]	Gaussian width [eV]	Lorentzian width [eV]	rel. area [%]
N	398.31	1.04	0.30	85.4
S	400.51	1.04	0.30	14.6
**Ar^+ (1 min) + 1.24 nm $CoPcF_{16}$**				
N	398.77	1.09	0.24	96.2
S	400.72	1.09	0.24	3.8

**f) Ar^+ (4 min) + 0.36 nm $CoPcF_{16}$**	Position [eV]	Gaussian width [eV]	Lorentzian width [eV]	rel. area [%]
N	398.34	1.02	0.26	86.2
S	400.35	1.02	0.26	13.8
**Ar^+ (4 min) + 0.88 nm $CoPcF_{16}$**				
N	398.72	1.26	1.26	91.2
S	400.74	0.10	0.10	8.8

Table S3: Fit parameters for the Mo 3d spectra measured with low surface sensitivity (hν = 900 eV) shown in Figure 5.

**a) MoS_2**	Position [eV]	Gaussian width [eV]	Lorentzian width [eV]	rel. area [%]
Mo(IV) $3d_{5/2}$	228.51	1.76	0.28	51.9
Mo(IV) $3d_{3/2}$	231.72	1.76	0.43	35.0
S 2s	225.64	2.00	0.67	13.1
**Ne^+ (1 min)**				
Mo(IV) $3d_{5/2}$	228.62	1.76	0.29	51.3
Mo(IV) $3d_{3/2}$	231.80	1.76	0.44	34.5
Mo(0) $3d_{5/2}$	227.56	1.76	0.28	0.7
Mo(0) $3d_{3/2}$	230.74	1.76	0.28	0.5
S 2s	225.79	2.00	0.68	13.0
**0.39 nm $CoPcF_{16}$**				
Mo(IV) $3d_{5/2}$	228.63	1.76	0.29	51.7
Mo(IV) $3d_{3/2}$	231.84	1.76	0.44	34.9
Mo(0) $3d_{5/2}$	227.05	1.75	0.28	0.7
Mo(0) $3d_{3/2}$	230.20	1.75	0.28	0.5
S 2s	225.76	2.00	0.62	12.1
**1.04 nm $CoPcF_{16}$**				
Mo(IV) $3d_{5/2}$	228.69	2.20	0.29	52.9
Mo(IV) $3d_{3/2}$	231.94	2.20	0.41	34.8
Mo(0) $3d_{5/2}$	227.40	2.10	0.29	0.8

Mo(0) $3d_{3/2}$	230.50	2.10	0.29	0.6
S 2s	225.63	2.00	0.68	10.9

**b) MoS_2**	Position [eV]	Gaussian width [eV]	Lorentzian width [eV]	rel. area [%]
Mo(IV) $3d_{5/2}$	229.02	1.76	0.20	50.7
Mo(IV) $3d_{3/2}$	232.21	1.76	0.30	34.6
S 2s	226.19	2.03	0.90	14.7
**Ar^+ (1 min)**				
Mo(IV) $3d_{5/2}$	228.69	1.76	0.20	51.1
Mo(IV) $3d_{3/2}$	231.90	1.76	0.30	34.7
S 2s	225.84	2.03	0.90	14.1
**0.19 nm $CoPcF_{16}$**				
Mo(IV) $3d_{5/2}$	228.71	1.76	0.20	50.5
Mo(IV) $3d_{3/2}$	231.88	1.76	0.30	34.4
S 2s	225.99	2.03	0.90	15.1
**1.24 nm $CoPcF_{16}$**				
Mo(IV) $3d_{5/2}$	228.64	1.76	0.20	50.7
Mo(IV) $3d_{3/2}$	231.79	1.76	0.30	34.2
S 2s	226.00	2.03	0.90	15.1

**c) MoS_2**	Position [eV]	Gaussian width [eV]	Lorentzian width [eV]	rel. area [%]

Mo(IV) $3d_{5/2}$	228.56	1.95	0.25	51.4
Mo(IV) $3d_{3/2}$	231.79	1.95	0.35	34.8
S 2s	225.65	1.50	1.29	13.8
**Ar^+ (4 min)**				
Mo(IV) $3d_{5/2}$	228.35	1.99	0.15	48.6
Mo(IV) $3d_{3/2}$	231.56	1.99	0.30	32.6
Mo(0) $3d_{5/2}$	227.53	2.00	0.15	2.8
Mo(0) $3d_{3/2}$	230.73	2.00	0.15	1.9
S 2s	225.49	2.00	0.88	14.1
**0.36 nm $CoPcF_{16}$**				
Mo(IV) $3d_{5/2}$	228.49	1.91	0.15	48.9
Mo(IV) $3d_{3/2}$	231.74	1.91	0.30	33.0
Mo(0) $3d_{5/2}$	227.30	2.00	0.15	3.3
Mo(0) $3d_{3/2}$	230.50	2.00	0.15	2.2
S 2s	225.55	1.73	1.00	12.5
**0.88 nm $CoPcF_{16}$**				
Mo(IV) $3d_{5/2}$	228.72	1.90	0.15	48.3
Mo(IV) $3d_{3/2}$	231.90	1.90	0.30	32.5
Mo(0) $3d_{5/2}$	227.28	2.00	0.15	3.6
Mo(0) $3d_{3/2}$	230.38	2.00	0.15	2.4
S 2s	225.89	1.75	1.00	13.3

Table S4: Fit parameters for the Mo 3d spectra measured with high surface sensitivity (hν =300 eV) shown in Figure 5.

**d) MoS_2**	Position [eV]	Gaussian width [eV]	Lorentzian width [eV]	rel. area [%]
Mo(IV) $3d_{5/2}$	228.81	0.15	0.18	51.0
Mo(IV) $3d_{3/2}$	231.97	0.15	0.41	31.8
S 2s	226.02	1.32	0.20	17.2
**Ne^+ (1 min)**				
Mo(IV) $3d_{5/2}$	228.86	0.32	0.20	45.7
Mo(IV) $3d_{3/2}$	232.03	0.32	0.40	29.1
Mo(0) $3d_{5/2}$	228.50	0.32	0.20	5.9
Mo(0) $3d_{3/2}$	231.55	0.32	0.20	3.8
S 2s	226.03	1.53	0.12	15.6
**0.39 nm $CoPcF_{16}$**				
Mo(IV) $3d_{5/2}$	228.93	0.32	0.20	47.6
Mo(IV) $3d_{3/2}$	232.10	0.32	0.44	31.4
Mo(0) $3d_{5/2}$	228.54	0.30	0.20	5.8
Mo(0) $3d_{3/2}$	231.74	0.30	0.20	3.7
S 2s	226.20	1.30	0.44	11.6
**1.04 nm $CoPcF_{16}$**				
Mo(IV) $3d_{5/2}$	228.87	0.31	0.20	45.8
Mo(IV) $3d_{3/2}$	232.04	0.31	0.44	30.7
Mo(0) $3d_{5/2}$	228.50	0.30	0.20	5.9
Mo(0) $3d_{3/2}$	231.62	0.30	0.20	3.8
S 2s	225.97	1.30	0.54	13.7

**e) MoS_2**	Position [eV]	Gaussian width [eV]	Lorentzian width [eV]	rel. area [%]
Mo(IV) $3d_{5/2}$	229.17	0.16	0.17	49.3
Mo(IV) $3d_{3/2}$	232.32	0.16	0.48	32.9
S 2s	226.19	0.80	1.0	17.8
**Ar^+ (1 min)**				
Mo(IV) $3d_{5/2}$	229.00	0.27	0.17	44.2
Mo(IV) $3d_{3/2}$	232.18	0.27	0.51	28.9
Mo(0) $3d_{5/2}$	228.63	0.27	0.18	6.8
Mo(0) $3d_{3/2}$	231.97	0.27	0.18	4.4
S 2s	226.14	0.80	1.00	15.7
**0.19 nm $CoPcF_{16}$**				
Mo(IV) $3d_{5/2}$	228.97	0.27	0.17	45.0
Mo(IV) $3d_{3/2}$	232.15	0.27	0.47	29.0
Mo(0) $3d_{5/2}$	228.63	0.27	0.18	6.9
Mo(0) $3d_{3/2}$	231.88	0.27	0.18	4.4
S 2s	226.11	0.80	1.00	14.7
**1.24 nm $CoPcF_{16}$**				
Mo(IV) $3d_{5/2}$	229.03	0.27	0.17	44.2
Mo(IV) $3d_{3/2}$	232.20	0.27	0.43	29.5
Mo(0) $3d_{5/2}$	228.70	0.27	0.18	6.5
Mo(0) $3d_{3/2}$	231.95	0.27	0.18	4.1
S 2s	226.12	0.80	1.00	15.76

**f) MoS_2**	Position [eV]	Gaussian width [eV]	Lorentzian width [eV]	rel. area [%]
Mo(IV) $3d_{5/2}$	228.95	0.22	0.14	50.9
Mo(IV) $3d_{3/2}$	232.11	0.22	0.38	32.7
S 2s	225.96	0.69	0.75	16.3
**Ar^+ (4 min)**				
Mo(IV) $3d_{5/2}$	228.85	0.41	0.14	43.8
Mo(IV) $3d_{3/2}$	232.02	0.41	0.38	28.4
Mo(0) $3d_{5/2}$	228.42	0.40	0.14	9.8
Mo(0) $3d_{3/2}$	231.61	0.40	0.14	6.5
S 2s	226.01	0.69	0.75	11.6
**0.36 nm $CoPcF_{16}$**				
Mo(IV) $3d_{5/2}$	228.83	0.41	0.14	43.4
Mo(IV) $3d_{3/2}$	232.03	0.41	0.38	28.0
Mo(0) $3d_{5/2}$	228.45	0.40	0.14	9.8
Mo(0) $3d_{3/2}$	231.68	0.40	0.14	6.5
S 2s	225.84	0.69	0.75	12.4
**0.88 nm $CoPcF_{16}$**				
Mo(IV) $3d_{5/2}$	228.90	0.42	0.14	41.9
Mo(IV) $3d_{3/2}$	232.12	0.42	0.39	27.3
Mo(0) $3d_{5/2}$	228.55	0.41	0.14	10.0
Mo(0) $3d_{3/2}$	231.77	0.41	0.14	6.5
S 2s	226.06	0.69	0.75	14.3

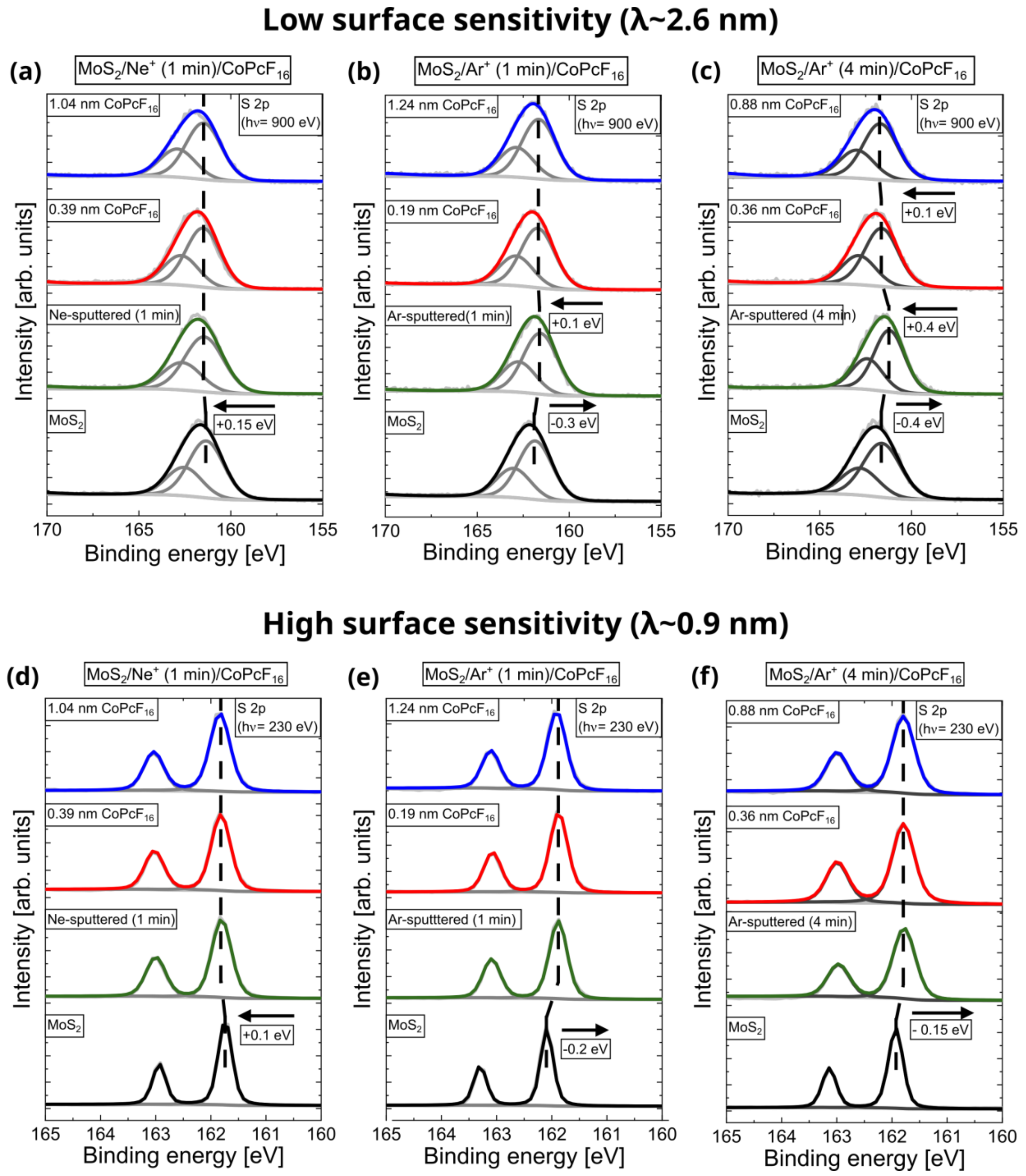

Figure S2: X-ray photoelectron spectroscopy (XPS) measurements of the S 2p core levels of MoS_2 following the sputtering with Ar and Ne and the deposition of $CoPcF_{16}$.

Table S5: Fit parameters for the S 2p spectra measured with low surface sensitivity (hν= 900 eV) shown in Figure S2.

**a) MoS_2**	Position [eV]	Gaussian width [eV]	Lorentzian width [eV]	rel. area [%]
S $2p_{3/2}$	161.33	2.14	0.20	66.7
S $2p_{1/2}$	162.53	2.14	0.20	33.3
**Ne^+ (1 min)**				
S $2p_{3/2}$	161.44	2.37	0.20	66.7
S $2p_{1/2}$	162.64	2.37	0.20	33.3
**0.39 nm $CoPcF_{16}$**				
S $2p_{3/2}$	161.50	2.10	0.20	66.7
S $2p_{1/2}$	162.70	2.10	0.20	33.3
**1.04 nm $CoPcF_{16}$**				
S $2p_{3/2}$	161.48	2.15	0.20	66.7
S $2p_{1/2}$	162.68	2.15	0.20	33.3

**b) MoS_2**	Position [eV]	Gaussian width [eV]	Lorentzian width [eV]	rel. area [%]
S $2p_{3/2}$	161.84	2.08	0.17	66.7
S $2p_{1/2}$	163.02	2.08	0.17	33.3
**Ar^+ (1 min)**				
S $2p_{3/2}$	161.57	2.08	0.17	66.7
S $2p_{1/2}$	162.75	2.08	0.17	33.3

**0.19 nm $CoPcF_{16}$**				
S $2p_{3/2}$	161.71	2.08	0.17	66.7
S $2p_{1/2}$	162.89	2.08	0.17	33.3
**1.24 nm $CoPcF_{16}$**				
S $2p_{3/2}$	161.65	2.08	0.17	66.6
S $2p_{1/2}$	162.87	2.08	0.17	33.4

**c) MoS_2**	Position [eV]	Gaussian width [eV]	Lorentzian width [eV]	rel. area [%]
S $2p_{3/2}$	161.61	2.26	0.24	66.7
S $2p_{1/2}$	162.81	2.26	0.24	33.3
**Ar^+ (4 min)**				
S $2p_{3/2}$	161.20	1.80	0.30	66.7
S $2p_{1/2}$	162.41	1.80	0.30	33.3
**0.36 nm $CoPcF_{16}$**				
S $2p_{3/2}$	161.63	2.00	0.32	66.6
S $2p_{1/2}$	162.87	2.00	0.32	33.4
**0.88 nm $CoPcF_{16}$**				
S $2p_{3/2}$	161.70	2.00	0.41	66.7
S $2p_{1/2}$	162.98	2.00	0.41	33.3

Table S6: Fit parameters for the S 2p spectra measured with high surface (hν= 230 eV) sensitivity shown in Figure S2.

**d) MoS_2**	Position [eV]	Gaussian width [eV]	Lorentzian width [eV]	rel. area [%]
S $2p_{3/2}$	161.74	0.23	0.10	68.6
S $2p_{1/2}$	162.94	0.23	0.10	31.4
**Ne^+ (1 min)**				
S $2p_{3/2}$	161.80	0.36	0.10	66.4
S $2p_{1/2}$	163.00	0.36	0.10	33.6
**0.39 nm $CoPcF_{16}$**				
S $2p_{3/2}$	161.81	0.36	0.10	65.8
S $2p_{1/2}$	163.03	0.36	0.10	34.2
**1.04 nm $CoPcF_{16}$**				
S $2p_{3/2}$	161.84	0.36	0.13	67.2
S $2p_{1/2}$	163.04	0.36	0.13	32.8

**e) MoS_2**	Position [eV]	Gaussian width [eV]	Lorentzian width [eV]	rel. area [%]
S $2p_{3/2}$	162.08	0.22	0.10	65.2
S $2p_{1/2}$	163.29	0.22	0.10	34.8
**Ar^+ (1 min)**				
S $2p_{3/2}$	161.89	0.33	0.10	66.5
S $2p_{1/2}$	163.08	0.33	0.10	33.5

**0.19 nm $CoPcF_{16}$**				
S $2p_{3/2}$	161.87	0.31	0.10	66.9
S $2p_{1/2}$	163.08	0.31	0.10	33.1
**1.24 nm $CoPcF_{16}$**				
S $2p_{3/2}$	161.91	0.34	0.10	68.1
S $2p_{1/2}$	163.11	0.34	0.10	31.9

**f) MoS_2**	Position [eV]	Gaussian width [eV]	Lorentzian width [eV]	rel. area [%]
S $2p_{3/2}$	161.94	0.26	0.10	67.6
S $2p_{1/2}$	163.13	0.26	0.10	32.4
**Ar^+ (4 min)**				
S $2p_{3/2}$	161.78	0.41	0.12	68.8
S $2p_{1/2}$	162.96	0.41	0.12	31.2
**0.36 nm $CoPcF_{16}$**				
S $2p_{3/2}$	161.79	0.36	0.19	66.3
S $2p_{1/2}$	162.99	0.36	0.19	33.7
**0.88 nm $CoPcF_{16}$**				
S $2p_{3/2}$	161.79	0.40	0.15	66.2
S $2p_{1/2}$	162.99	0.40	0.15	33.8

To give further insights in the nature of defects produced by sputtering, the change in Mo/S-ratio can be considered. The ratio is calculated with the core level intensities given in Table S1 and the photoionization cross-sections σ provided by Yeh and Lindau [4] via Equation S1.

$$\text{Mo:S}=\frac{I_{\text{Mo 3d}}/\sigma_{\text{Mo 3d}}}{I_{\text{S 2s}}/\sigma_{\text{S 2s}}} \quad \text{(S1)}$$

Table S7: Mo/S-ratio before and after sputtering for the experiments depicted in Figure 5.

Experiment	Mo:S (before sputtering)	Mo:S (after sputtering)	Relative change (%)
Ne^+ (1 min, hν= 900 eV)	1:1.56	1:1.55	-0.1
Ar^+ (1 min, hν= 900 eV)	1:1.79	1:1.70	-5.3
Ar^+ (4 min, hν= 900 eV)	1:1.66	1:1.70	+2.4
Ne^+ (1 min, hν= 300 eV)	1:2.89	1:2.57	-12.5
Ar^+ (1min, hν= 300 eV)	1:3.01	1:2.59	-16.2
Ar^+ (4min, hν= 300 eV)	1:2.71	1:1.83	-48.1

References

[1] Belser, A.; Greulich, K.; Grüninger, P.; Karstens, R.; Ovsyannikov, R.; Giangrisostomi, E.; Nagel, P.; Merz, M.; Schuppler, S.; Chassé, T.; Peisert, H. Perfluorinated Phthalocyanines on Cu(110) and Cu(110)-(2 × 1)O: The Special Role of the Central Cobalt Atom. *J. Phys. Chem. C* **2021**, *125* (16), 8803–8814. https://doi.org/10.1021/acs.jpcc.1c01215.

[2] Haizmann, P.; Juriatti, E.; Klein, M.; Greulich, K.; Ovsyannikov, R.; Giangrisostomi, E.; Chassé, T.; Peisert, H.; Scheele, M. Tuning the Interfacial Electronic Structure of MoS2 by Adsorption of Cobalt Phthalocyanine Derivatives. *ACS Appl. Electron. Mater.* **2024**, *6* (4), 2467–2477. https://doi.org/10.1021/acsaelm.4c00094.

[3] Peisert, H.; Kolacyak, D.; Chassé, T. Site-Specific Charge-Transfer Screening at Organic/Metal Interfaces. *J. Phys. Chem. C* **2009**, *113* (44), 19244–19250. https://doi.org/10.1021/jp9057548.

[4] Yeh, J. J.; Lindau, I. Atomic Subshell Photoionization Cross Sections and Asymmetry Parameters: 1 ⩽ Z ⩽ 103. *Atomic Data and Nuclear Data Tables* **1985**, *32* (1), 1–155. https://doi.org/10.1016/0092-640X(85)90016-6.
